# When the weather outside is frightful, let it condensate: How the phase separation of an RNA binding protein CP29A helps plants acclimatize to cold

**DOI:** 10.1093/plcell/koae157

**Published:** 2024-05-24

**Authors:** Sonhita Chakraborty

**Affiliations:** Assistant Features Editor, The Plant Cell, American Society of Plant Biologists; Department of Forest Genetics and Plant Physiology, Swedish University of Agricultural Sciences, Umeå Plant Science Centre, Umeå, Sweden

Biology textbooks readily inform us about the importance of membrane-bound organelles like mitochondria, Golgi apparatus, and the vacuole, but what about “organelles” that lack membranes? The liquid-liquid phase separation (LLPS) of proteins and RNA into “membrane-less organelles,” also known as biomolecular condensates, is dynamic and reversible, depending on factors such as salt concentration, temperature, and protein levels. While much of the seminal work on biomolecular condensates has been conducted in animal systems, plant biologists too are starting to realize that plant cellular components form condensates (Field et al. 2023; and see other articles in *The Plant Cell* July 2023 focus issue on biomolecular condensates, introduced by Gutierrez-Beltran et al. 2023).

Much like their mammalian counterparts, plant proteins rely on special domains like Intrinsically Disordered Regions (IDRs) to form condensates in response to environmental stresses and developmental cues (Field et al. 2023). Understanding the molecular mechanisms of the assembly, interaction with cellular components, and the subsequent dissociation of highly dynamic condensates will help to clarify how rapid changes occur in the cell. In recent years, the dynamic assembly of biomolecular condensates in response to environmental stressors has been reported in several publications (Field et al. 2023). Regarding temperature stress, most temperature-induced condensates have been observed in the nucleus (Jung et al. 2020; Zhang et al. 2023). Now, Legen and coauthors (2024) provide evidence of cold-induced LLPS in the chloroplast.

As its name suggests, CHLOROPLAST RNA-BINDING PROTEIN 29A (CP29A) is a chloroplast-localized protein that binds RNA. Without functional CP29A, plants exhibit bleached de novo tissues and defective chloroplast RNA processing and accumulation under cold conditions (Kupsch et al. 2012). In their current work, the authors find that under cold stress, *cp29a* mutants display aberrant intron splicing not just after long-term but also after short-term cold acclimation. This is interesting given that despite these splicing defects, neither long-term nor short-term exposure to cold led to global changes in chloroplast RNA abundance, as demonstrated by ribosome profiling and RNA-sequencing experiments in wild type and *cp29a*. The authors found that a predicted prion-like domain (PLD) of CP29A is important for splicing efficiency as *CP29AΔPLD* mutant plants could only partially complement chloroplast RNA splicing defects under short-term cold acclimation (Legen et al. 2024). In the same vein, the *CP29AΔPLD* mutant plants could not adapt at 8 °C and showed pale discoloration, a phenotype that was complemented by transgenic expression of full-length CP29A (Figure.). This demonstrated the importance of CP29A and its PLD in adaptation to low temperatures and RNA processing.

**Figure.**
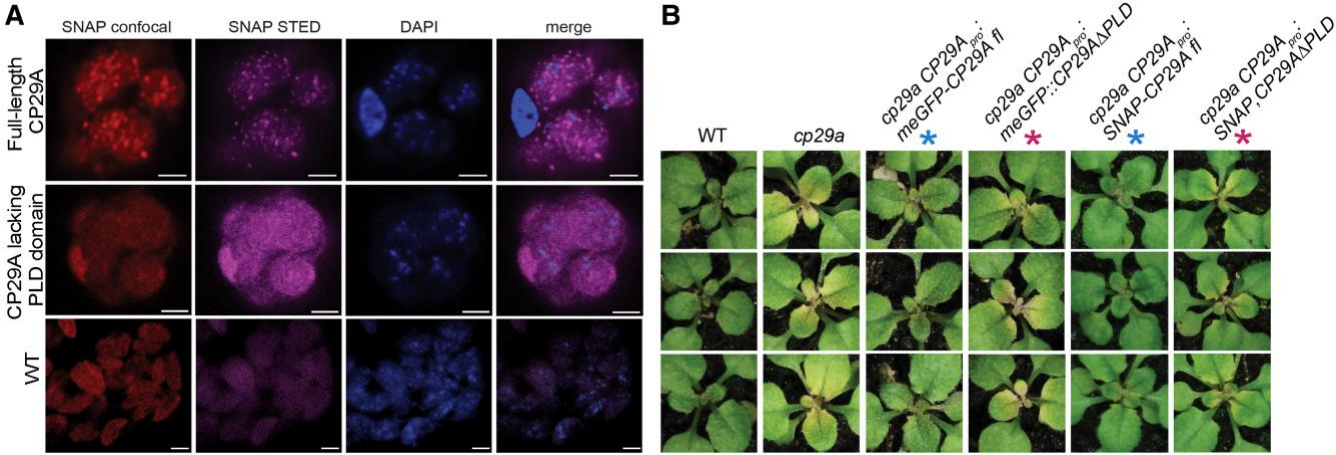
CP29A's PLD domain is important for its function in cold resistance and its ability to phase separate. **A)** Confocal and stimulated emission depletion microscopy images of SNAP-tagged wild-type (WT), CP29Apro:SNAP-CP29Afl (full-length CP29A), and CP29Apro:SNAP-CP29AΔPLD (CP29A lacking PLD domain) protoplasts exposed to 1 h of cold treatment (8°C) show that full-length CP29A including its PLD is required for its cold-induced phase separation and formation of granular bodies. **B)** Full-length CP29A (tagged with GFP or SNAP) is required to complement the pale tissue of the *cp29a* null mutant (rows marked with a blue asterisk) as the CP29AΔPLD expressing plants (rows marked with a magenta asterisk) were unable to do so. Adapted from Legen et al. (2024), Figures 3 and 4E.

The importance of the PLD of CP29A extends further within the cell. The PLD was found to be essential for CP29A to undergo phase separation in vitro and in plant protoplasts upon cold stress acclimation (Figure.) (Legen et al. 2024). Recombinant proteins lacking a PLD (*CP29AΔPLD*) were unable to properly undergo LLPS or reversible fluorescence recovery after photobleaching. Stimulated emission depletion microscopy demonstrated that the condensates were situated adjacent to nucleoids. This might indicate that chloroplasts make use of an RNA processing compartment next to transcribed organellar DNA, similar to what was observed for human mitochondria (Antonicka et al. 2013; Jourdain et al. 2016). Indeed, chloroplast RNAs were found to be incorporated into the CP29A condensates, in line with its function as an RNA binding protein (Legen et al. 2024). These experiments supported the requirement of PLD in facilitating CP29A droplet formation when exposed to cold temperatures. It also draws attention to the intricate compartmentalization that occurs in the chloroplast, the relevance of which might extend beyond RNA metabolism.

Besides characterizing CP29A's function in cold resistance, Legen et al. (2024) noticed that CP29A forms irreversible stress granules upon heat treatment. Given that the protein was identified from a recent proteomic study of heat-induced chloroplast condensates (Chodasiewicz et al. 2020), it would be interesting to dissect if CP29A plays a larger role in temperature sensing and integration for both heat and cold stressors and the separation of function in these responses. The temperature-dependent condensate formation of an RNA-binding protein in the chloroplast raises questions about its exact function in RNA processing and splicing, all ultimately important as we inch closer to understanding the effects of climate change on our green planet.

## Dive Curated Terms

The following phenotypic, genotypic, and functional terms are of significance to the work described in this paper:
